# Differential Nrf2 expression between glioma stem cells and non-stem-like cells in glioblastoma

**DOI:** 10.3892/ol.2013.1760

**Published:** 2013-12-16

**Authors:** JIANHONG ZHU, HANDONG WANG, XIANGJUN JI, LIN ZHU, QING SUN, ZIXIANG CONG, YUAN ZHOU, HUANDONG LIU, MENGLIANG ZHOU

**Affiliations:** 1 Department of Neurosurgery, Jinling Hospital Affiliated to Nanjing University School of Medicine, Nanjing, Jiangsu 210089, P.R. China; 2 Department of Neurosurgery, Jinling Hospital, Neurosurgical Institution of People’s Liberation Army of China, Nanjing, Jiangsu 210002, P.R. China; 3 Neurosurgery Department of Southern Medical University, Guangzhou, Guangdong 510515, P.R. China

**Keywords:** glioblastoma, glioma stem cell, nuclear factor erythroid 2-related factor 2

## Abstract

Glioblastoma multiforme (GBM), the most commonly occurring primary intracranial tumor, is associated with a negative outcome, regardless of the availability of multimodal therapies. However, the identification of glioma stem cells (GSCs), which are small groups of cells within the GBM, has resulted in novel avenues for research. GSCs are resistant to numerous types of environmental stress, such as irradiation, antitumor drugs and hypoxia. Nuclear factor erythroid 2-related factor 2 (Nrf2) has a significant role the cellular response to oxidative stress and previous studies have supported the significance of Nrf2 in GBM; however, the role of Nrf2 in GSCs remains unclear. In the present study, Nrf2 in CD133^−^ GBM cells and CD133^+^ GSCs from GBM were compared. GSCs from GBM, which express the surface marker CD133, were separated by magnetic cell sorting and analyzed by immunofluorescence in 24-well clusters and cell counting using flow cytometry. The expression of Nrf2 was detected at the transcriptional and translational levels in CD133^+^ and CD133^−^ cells, and the result indicated that GSCs were successfully isolated from the GBM. The percentage of tumor stem cells in total cells was between 0.49 and 0.91%. Nrf2 was overexpressed in CD133^+^ GSCs when compared with CD133^−^ GBM cells, which indicated that the expression of Nrf2 in GSCs was closely correlated with malignant proliferation and differentiation of the GBM. Therefore, it was concluded that Nrf2 may be a potential biomarker and rational therapeutic target in GBM.

## Introduction

Glioblastoma multiforme (GBM) is a malignant tumor that responds poorly to radiotherapy and chemotherapy (1). Glioma stem cells (GSCs) are a small percentage of glioma cells that demonstrate features of primitive neural progenitor cells and tumor-initiating functions (2). It is hypothesized that GSCs promote tumor progression in addition to being possible ‘seeds’ of recurrence following conventional therapies for GBM (3,4). GSCs are resistant to current therapeutic methods, including radiotherapy, chemotherapy and anti-angiogenesis therapy (5–7), and their capacity for anti-hypoxia is greater than that of other cells in the GBM (8). In addition, self-renewal and the undifferentiated state of GSCs are known to be enhanced by hypoxia (9,10); however, the underlying mechanisms have not been fully identified.

Nuclear erythroid 2-related factor 2 (Nrf2) is a redox-sensitive, basic leucine zipper protein that regulates the transcription of certain antioxidant genes. It is a key nuclear transcription factor, which regulates antioxidant response element (ARE)-containing genes (11). Furthermore, Nrf2 constitutes a predominant detoxification system in numerous types of cell (12,13) and has been implicated in cancer prevention (13,14), for example in GBM (15). Therefore, we hypothesized that Nrf2 is significant within GSCs; however, the expression and the role of Nrf2 in the anti-hypoxia action of GSCs remains unclear.

Thus, the levels of Nrf2 in transcription and translation were analyzed in the present study, and the transcription of mRNA and the translation of proteins in GSCs was investigated using real-time polymerase chain reaction (qPCR) and western blot analysis. As a result, it was hypothesized that Nrf2 was significant in GSC resistance to environmental stress, specifically in anti-hypoxia and metabolism therapies, which may be beneficial to future studies. Therefore, Nrf2 may be a potential target for the treatment of GBM.

## Materials and methods

### Ethical approval

The present study was conducted in accordance with the ethics committee of Jinling Hospital (Jiangsu, China). All patients provided informed written consent for involvement in this study.

### Cell direction and treatment

Primary human GBM cells (G1, G2 and G3) were derived from freshly resected human surgical GBM specimens, which were obtained from three patients at the Department of Neurosurgery in Jinling Hospital. The samples were identified as GBM World Health organization grade IV by the pathologists at Jinling Hospital. The tumors were digested with collagenase type IV (Sigma-Aldrich, St. Louis, MO, USA) and released to single cells by gentle pipetting, and then filtered through a 70-μm cell strainer. The adherent culture of GBM cells were seeded in Dulbecco’s modified Eagle’s medium with Ham’s F12 medium (DMEM/F-12; Gibco-BRL, Carlsbad, CA, USA) containing 10% fetal bovine serum (FBS; Hyclone, Waltham, MA, USA) with a density of 2×10^5^ live cells/ml. After 15 min, the nonadherent cells were seeded in DMEM/F-12 containing 10% FBS at a density of 2×10^5^ live cells/ml.

### Flow cytometry

Fluorescence-activated cell sorting (FACS) was performed to evaluate the number of CD133^+^ cells. The GBM cells were collected and washed in phosphate-buffered saline (PBS) three times and incubated with CD133-phycoerythrin (Miltenyi Biotec, Gladbach, Germany) at 37°C for 40 min in a humidified chamber, followed by an additional wash using PBS. The labeled cells were analyzed using a BD FACSAria^™^ III system (BD Biosciences, Franklin Lakes, NJ, USA). The data were analyzed using FlowJo 7.6 software (Tree Star, Inc., Ashland, OR, USA).

### Magnetic cell sorting (MACS) and cell culture

MACS was conducted as described previously (16). Cells were dissociated and resuspended in PBS containing 0.5% bovine serum albumin and 2 mmol/l EDTA. CD133 MicroBeads (Miltenyi Biotec) were used for magnetic labeling and MACS was performed with the MiniMACS machine (Miltenyi Biotec). Positive magnetic cells were separated using several MACS columns in series.

The majority of the CD133^+^ cells (GSC1, obtained from G1; GSC2, obtained from G2 and GSC3, obtained from G3) were harvested for protein assaying and refinement of mRNA. The remaining cells were used for immunofluorescence analysis.

The CD133^−^ cells were seeded in DMEM/F-12 containing 10% FBS at a density of 2×10^5^ live cells/ml. The cells were maintained in a standard tissue culture incubator at 37°C with 5% CO_2_ in air and 100% relative humidity.

### Contribution of GSC spheres

A small quantity of CD133^+^ cells were cultured in serum-free DMEM/F-12, in addition to a neural supplement from the Neural Stem Cell kit (Invitrogen Life Technologies, Carlsbad, CA, USA), 20 ng/ml recombinant human epidermal growth factor (Invitrogen Life Technologies), 20 ng/ml recombinant human basic fibroblast growth factor (Invitrogen Life Technologies), 100 IU/ml penicillin G and 100 μg/ml streptomycin; this step identified the generation of GSC spheres.

### Immunofluorescence analysis of GSCs

CD133^+^ and CD133^−^ cells were incubated in DMEM/F-12 containing 10% FBS for 4 h. The cells were fixed in 4% paraformaldehyde (PFA) and incubated at 4°C with a mouse polyclonal primary antibody against nestin (1:500; Abcam, Cambridge, UK) and a rabbit polyclonal primary antibody against Nrf2 (1:500; Abcam), according to the manufacturer’s instructions.

For identifying the generation of the GSC spheres, certain CD133^+^ cells were cultured in neural stem cell medium, fixed in 4% PFA and incubated at 4°C with a rabbit polyclonal primary antibody against CD133 (1:500; Biorbyt, Cambridge, UK).

After 12 h, Dylight 594-AffiniPure goat anti-rabbit secondary antibody (red fluorescence; 1:100; EarthOx LLC, San Francisco, CA, USA) and Dylight 488 AffiniPure goat anti-mouse secondary antibody (green fluorescence; 1:100; EarthOx) were added and incubated for 2 h at room temperature. The cells were mounted with mounting media containing 4′,6-diamino-2-phenylindole (Vector Laboratories, Burlingame, CA, USA) and were observed under a fluorescence microscope (Axio Observer A1; Carl Zeiss, Oberkochen, Germany).

### Western blot analysis

Western blot analysis was performed on the protein isolated from the cell lysates of CD133^+^ and CD133^−^ GBM cells (GSC1, GSC2, GSC3 and G1, G2, G3, respectively) using the rabbit polyclonal antibodies against Nrf2 (1:500), the rabbit polyclonal antibodies against nestin (1:500) and the rabbit polyclonal antibodies against glial fibrillary acidic protein (1:1,000). Nestin is an important GSC marker (17); therefore, antibodies against nestin were used to indicate the stem cell characteristics of CD133^+^ cells. The blots were stripped and reprobed with anti-GAPDH (rabbit polyclonal; Bio-World, Dublin, OH, USA) to determine the equivalent loading, as described in previous studies (18). The cells were lysed in lysis buffer that comprised of 50 mM Tris-HCl, pH 7.5; 150 mM NaCl, 0.5% TX-100, 5% glycerol, 1% SDS, 1 mM Na_3_VO^4^, 10 mM NaF and 1 mM phenylmethanesulfonyl fluoride. The protein concentrations were determined using a DC Protein assay (Bio-Rad, Hercules, CA, USA) and 5X SDS sample buffer (0.5 M Tris-HCl, pH 6.8; 28% glycerol, 9% SDS, 5% 2-mercaptoethanol and 0.01% bromphenol blue) was added. The lysates were electrophoresed on an 8% SDS-PAGE gel and transferred to a nitrocellulose membrane (Merck Millipore, Darmstadt, Germany). The membranes were incubated with primary antibodies at 4°C overnight. The horseradish peroxidase-conjugated secondary antibodies were exposed to radiographic film (Kodak Film, Rochester, NY, USA) using an enhanced chemiluminescence reagent (Merck Millipore).

### RNA preparation and qPCR

Total RNA was prepared using TRIzol (Takara Bio, Inc., Shiga, Japan), according to the manufacturer’s instructions. Approximately 400 ng total RNA was used as a template for cDNA synthesis using the Verso cDNA synthesis kit (Takara Bio, Inc.). qPCR was conducted using a SYBR^®^-Green Master mix (Takara Bio, Inc.), according to the manufacturer’s instructions. qPCR was conducted using the ABI 7900HT sequence detection system (Applied Biosystems, Foster City, CA, USA). The primers of the target genes are listed in Table. I, which indicates the amplicon length and annealing temperature.

### Statistical analysis

The values are supplied as the mean ± SD. The data were analyzed for significance using Student’s t-test. The differences between the groups were analyzed using analysis of variance followed by a post hoc test using the SPSS 19.0 package (SPSS Inc., Chicago, IL, USA). P<0.05 was considered to indicate a statistically significant difference.

## Results

### GSCs in GBM

**Figure 1:**
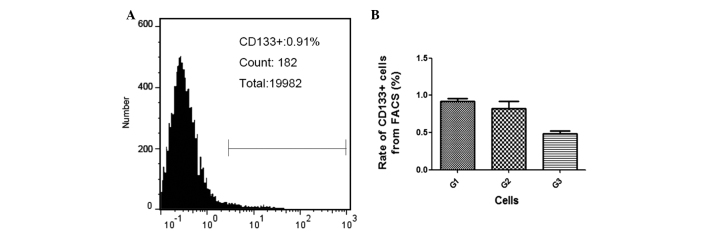
The ratio of CD133^+^ glioma stem cells in human GBM was determined using flow cytometry. (A) Experimental group incubated with CD133/2-phycoerythrin. (B) The rate of CD133^+^ cells from the GBM of three patients (G1, G2 and G3). FACS, fluorescence-activated cell sorting; GBM, glioblastoma multiforme.

In the present study, the ratio of CD133^+^ to CD133^−^ cells in GBM was analyzed using a flow cytometry assay. The GBM samples (GSC1, GSC2 and GSC3) contained ~0.91±0.07%, 0.83±0.11% and 0.49±0.06% CD133^+^ cells, respectively, under the culture condition of a 5% CO_2_ atmosphere (Fig. 1); this result was similar to that of previous studies (19).

### Culture of GSC spheres

**Figure 2:**
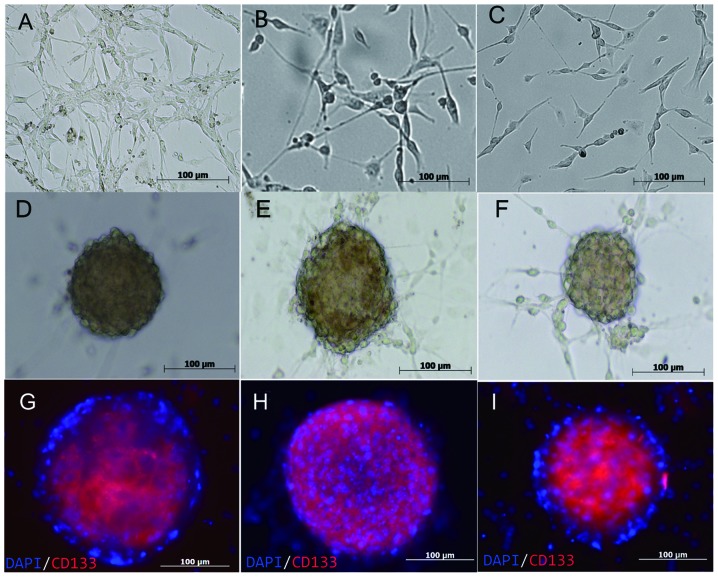
Morphology of GBM cells and GSCs. (A) G1, (B) G2 and (C) G3 GBM cells. (D, E and F) GSC spheres dissected from GBM cells (GSC1, GSC2 and GSC3, respectively). (G, H and I) CD133^+^ GSCs (red) dissected from the GBM. GBM, glioblastoma multiforme; GSC, glioma stem cell.

The GSC spheres were cultured in non-adherent growth, serum-free neural stem cell medium and spheres formed after two days (Fig. 2). Following ~10 days of rapid growth, the cell spheres attained a diameter of ~100 μm.

### Immunofluorescence of CD133^+^ GSCs and CD133^−^ cells

Nrf2 was significantly expressed in CD133^+^ GBM cells, regardless of which patient they were derived from. The CD133^+^ and CD133^−^ cells from GBM were cultured in the same medium for ~4 h, which did not significantly influence the differentiation of GSCs.

**Figure 3:**
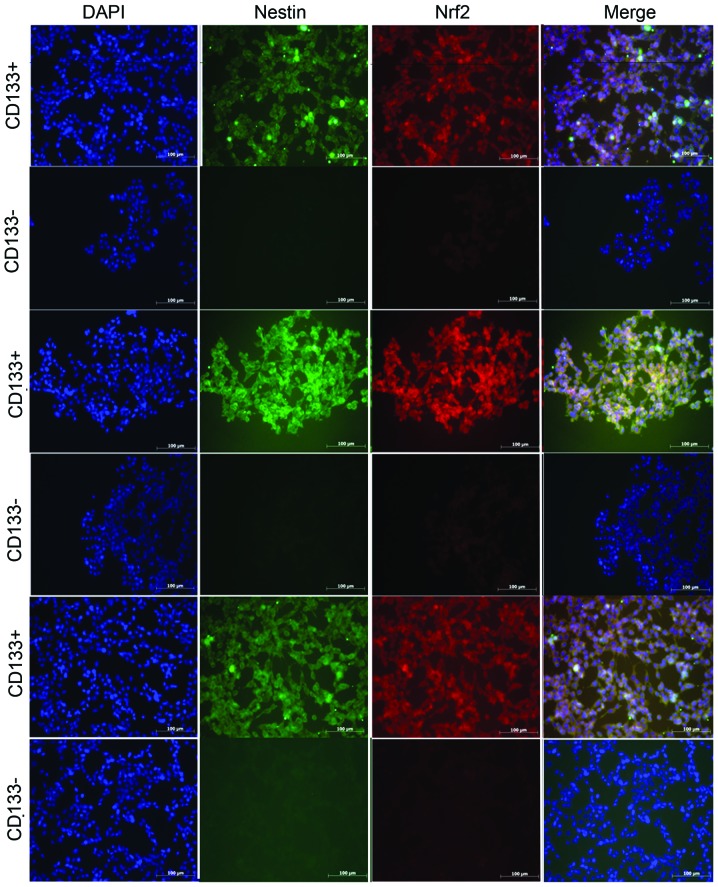
Immunoassays of the tumor cell in different media. CD133 was conjugated with green secondary antibody, Nrf2 was conjugated with red secondary antibodies and 4′,6-diamino-2-phenylindole highlighted the nucleus in blue. Nrf2 was apparent in the nucleus, identified by the red staining.

Conversely, GSC spheres grew rapidly in the serum-free medium. The present study, therefore, demonstrated that the majority of the cells, which were derived from MACS, expressed the neural stem cell biomarker CD133 (Fig. 3) (20,21) and were capable of cultivating GSC spheres.

### Nrf2 expression in CD133^−^ cells and CD133^+^ GSCs at the transcriptional level

**Figure 4:**
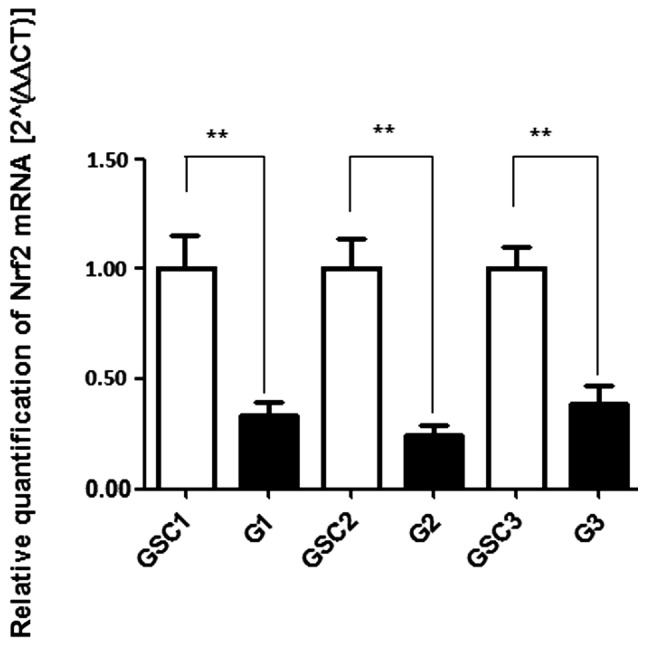
Real-time polymerase chain reaction analysis identified that the mRNA of Nrf2 was transcribed at varying levels. The expression of Nrf2 was greater in CD133^+^ cells (GSC1, GSC2 and GSC3) compared with that in the CD133^−^ cell lines (G1, G2 and G3), respectively.^**^P<0.05.

qPCR was conducted to assess the relative quantities of Nrf2 mRNA in CD133^+^ GSCs and CD133^−^ GBM cells. The qPCR data identified that the expression of Nrf2 was greater in CD133^+^ GSCs than that observed in CD133^−^ GBM cells (P<0.05; Fig. 4).

### Western blot analysis

**Figure 5:**
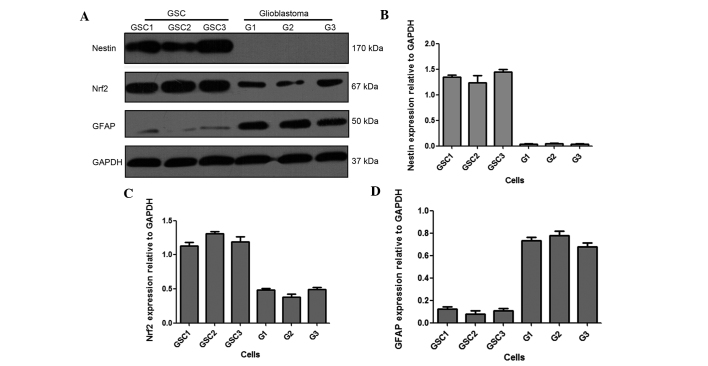
Western blot analysis showing the Nrf2 expression. Cell lysates were subjected to western blot analysis using antibodies specific to Nrf2; an antibody for GAPDH was used as a control. (A) Western blot analysis results. (B, C and D) The expression level was assessed via densitometry analysis. Nrf2 in CD133+ GSCs showed an increased expression compared with that in CD133- GBM cells, and only the GSCs expressed nestin. The GFAP levels in CD133- GBM cells were significantly higher than that in the CD133+ GSCs. Nrf2, nuclear erythroid 2-related factor 2; GFAP, glial fibrillary acidic protein; GAPDH, glyceraldehyde-3-phosphate dehydrogenase; GSC, glioma stem cell; GBM, glioblastoma multiforme.

Western blot analysis was conducted and a similar difference in expression levels was observed. The western blot analysis showed the total Nrf2 protein was expressed to a greater extent in CD133^+^ GSCs compared with that in CD133^−^ GBM cells (Fig. 5).

## Discussion

GBM is considered to be the central nervous system tumor with the highest rate of malignancy (22) and numerous studies have identified that GSCs are a significant factor (23,24). Furthermore, previous studies have identified that Nrf2 is important in GBM genesis *in vivo* and *in vitro* (15,25). As Nrf2 was hypothesized to be significant in GSC and tumor genesis, it was necessary to understand whether transcription and translation rates varied in GSCs (26).

The present study required a stable and reliable source of GSCs for analysis. Three predominant sources of GSCs have been identified: The tissue of GBM patients (3), cell lines (27) or xenografts of nude mice. In our preliminary experiments, there were complications with obtaining GSCs from GBM cell lines using the neural stem cell medium. Previous studies have adopted the MACS system to extract GSCs from GBM cell lines (16,27). Thus, MACS was conducted in the present study to obtain pure, stable GSCs from the GBM. CD133 and nestin antibodies were used for cell identification (21,28).

The transcriptional and translational protein levels observed in the present study demonstrated that the expression of Nrf2 in CD133^+^ GSCs was significantly higher than that in CD133^−^ cells (from the GBM). Western blot analysis suggested that the quantity of Nrf2 in CD133^−^ GBM cells was lower than that in CD133^+^ GSCs. This was confirmed by qPCR, as the expression of Nrf2 in CD133^+^ GSCs was greater than that observed in CD133^−^ GBM cells (P<0.05). Immunofluorescence and immunocytochemical analysis demonstrated a high level of Nrf2 in CD133^+^ GSCs, which was observed in the nucleus as well as the cytoplasm (Fig. 3). Nrf2 was capable of regulating ARE following its translocation into the nucleus. Numerous studies have identified that GSCs were more resistant to hypoxia and anti-angiogenesis therapies compared with CD133^−^ GGM cells (22); furthermore, hypoxia induces the invasion of GSCs into the underlying tissue (8). Nrf2 is one of the primary transcriptional regulators of anti-hypoxic and antioxidant reactions, which alter gene transcription following translocation into the nucleus and connected to the ARE region. The present study identified an increased expression of Nrf2 in GSCs; therefore, it was hypothesized that Nrf2 was critical in GSC genesis. However, this may be a result of the increased expression of an anti-hypoxia gene, as well as additional genes and molecules, which contribute to cell proliferation, differentiation, apoptosis, necrosis, self-renewal and the cell cycle (14,29). Thus the present study indicated that Nrf2 may be a potential biomarker and rational therapeutic target for the diagnosis and treatment of GSCs.

However, there were limitations in the present study; the influence of Nrf2 on GSC genesis, self-renewal and differentiation was not investigated. Although immunofluorescence assays identified that Nrf2 translocated into the cell nucleus, the associated genes regulated by this nuclear factor, such as quinone oxidoreductase1 and hemoxygenase-1 (30), were not analyzed; these may be key genes within GSCs. Furthermore, the Nrf2 expression was only assayed *in vitro* and as the environment within humans is complex, the interaction of cells and tissue fluid *in vivo* may alter the expression of Nrf2. Therefore, further study is required.

The mechanism behind the variable Nrf2 levels that exist between the GSCs and other GBM cells requires further investigation. In addition, the Nrf2 signaling pathway may be a novel target for the regulation and suppression of the proliferation and invasion of GSCs into the underlying tissue. In conclusion, Nrf2 may be considered as a potential target for the treatment of tumors in humans.

## Figures and Tables

**Table I:** Primers of target genes.

Gene	Primer sequence	Temperature (°C)	Length (bp)	Amplicon size (bases)
Nrf2	F: 5′-TCAGCGACGGAAAGAGTATGA-3′	60.6	21	174
	R: 5′-CCACTGGTTTCTGACTGGATGT-3′	61.9	22	
GAPDH	F: 5′-GAAATCCCATCACCATCTTC-3′	59.6	20	226
	R: 5′-CCACTGGTTTCTGACTGGATGT-3′	61.3	22	

Nrf2, nuclear erythroid 2-related factor 2.

## Abbreviations

GSCs: glioma stem cells
GBM: glioblastoma multiforme
Nrf2: nuclear factor erythroid 2-related factor 2
ARE: antioxidant response element
